# Effects and potential pathways of goose astrovirus infection on gosling hepatic lipid metabolism

**DOI:** 10.3389/fmicb.2025.1531373

**Published:** 2025-02-25

**Authors:** Chao Yin, Yun Shi, Haiqin Li, Zhihua Lu, Xiaona Gao, Guoliang Hu, Xiaoquan Guo

**Affiliations:** ^1^Jiangxi Provincial Key Laboratory for Animal Health, Institute of Animal Population Health, College of Animal Science and Technology, Jiangxi Agricultural University, Nanchang, Jiangxi, China; ^2^Institute of Animal Husbandry and Veterinary Medicine, Jiangxi Academy of Agricultural Sciences, Nanchang, Jiangxi, China

**Keywords:** astrovirus, gosling, infection, lipid metabolism, liver, pathway

## Abstract

**Introduction:**

The adverse effects of goose astrovirus (GoAstV) on avian growth and health have been widely reported previously, while the stress reactions and corresponding mechanism of gosling liver responding to GoAstV infection remain not entirely clear.

**Methods:**

One-day-old goslings inoculated subcutaneously with 2 × 10^−6^ TCID_50_ of GoAstV were employed as an experimental model, and the potential effects and pathways of GoAstV infection on gosling liver functions were investigated by combining the morphological, biochemical and RNA sequencing (RNA-seq) techniques.

**Results:**

Structural and functional impairments were found in gosling livers post the virus infection, as characterized by the histological alterations in liver index and morphology of hepatic cord and sinuses, as well as the abnormal expression patterns of the cellular antioxidant, inflammation and apoptosis-related genes. RNA sequencing analysis were performed to investigate the underlying mechanism. Results showed that the analysis of screened 1949 differentially expressed genes (DEGs) were mainly enriched in GO terms related to organic immune defense and substance metabolism, and their corresponding KEGG pathways represented by PPAR signaling pathway, intestinal immune network for IgA production, and fatty acid metabolism and degradation, suggesting that the functions of avian immunity and lipid metabolism were greatly changed after the GoAstV infection. Finally, the lipid deposition in gosling hepatocytes were further demonstrated by the subsequent Oil red O staining, biochemical detection of serum TG and HDL-C, and the gene expression analysis including *PPARα*, *PPARγ*, *ACSBG2*, *ACSL5*, *CPT1A* and *PCK1*.

**Discussion:**

Though limitations exist, the findings of this study are helpful to expand our understanding about the negative effects of GoAstV on goslings, and provide us with new clues for the salvaging of GoAstV-induced liver dysfunctions in poultry industry.

## 1 Introduction

Goose astrovirus (GoAstV) is a single-stranded RNA virus that mainly causes inflammation and gout in the viscera and joints of goslings up to 3 weeks of age (An et al., 2020; Cortez et al., 2017). Since its first discovery in China in 2016, the virus has spread rapidly to major goose-producing provinces throughout the country, causing a pathogenic rate as high as 80% and a fatality rate of 50% in goslings (Zhu et al., 2022). Furthermore, like most RNA viruses, GoAstV lacks the proofreading capability during its nucleic acid replication process, leading to high genetic diversity and recombination potential (Holland et al., 1982). As a result, GoAstV not only spreads among goose populations (Wei et al., 2020a) but also infects different species, including chickens and ducks (Li et al., 2021; Wei et al., 2020b). This widespread transmission has led to substantial losses in the poultry-breeding industry in China (Liu et al., 2022). However, compared to previous studies on genetic variation and pathogenicity, less attention has been given to the pathogenesis and therapeutic analysis of GoAstV until now.

Excessive production and excretion obstruction of uric acid (**UA**), as well as functional damage to visceral organs, have been reported to be two crucial factors in diseases induced by GoAstV in poultry (Wu et al., 2020; Yin et al., 2021). Among these, the liver not only serves as a vital organ for energy and material metabolism (Laliotis et al., 2010) but also is the primary site of UA production in poultry (Dalbeth et al., 2016; Maiuolo et al., 2016). For example, when goslings are infected with viruses such as GoAstV, UA production in their livers increases dramatically, leading to urate deposition in the blood, internal organs, and joint cavities, which subsequently causes hyperuricemia and gout (Xu et al., 2023; Yin et al., 2021). At the same time, the occurrence of cell apoptosis, oxidative stress, inflammation, and growth restriction in animals is also induced, as indicated by abnormal changes in body weight and the expression of cytokines such as caspase-3, superoxide dismutase (SOD), and nuclear factor kappa B (NF-κB) (Hou et al., 2024; Li Y et al., 2024). However, to date, the reactions and corresponding regulatory mechanisms of gosling livers in response to GoAstV infection are still not fully understood. Therefore, by combining morphological, biochemical, and RNA sequencing (RNA-seq) techniques, the present study aimed to investigate the effects of GoAstV infection on gosling liver function and uncover the potential regulatory pathways involved, synchronously. The results of this study enhance our understanding of the negative effects of GoAstV on goslings and provide new insights for addressing GoAstV-induced liver dysfunction in the poultry industry.

## 2 Materials and methods

### 2.1 Animals and experimental design

A total of 200 unvaccinated, GoAstV-negative Xingguo Gray geese were purchased from a commercial hatchery (Guohua Co. Ltd., Nanchang, China) at 1 day of age and raised in shielded cages with positively filtered air throughout the entire experimental process. Upon arrival, all goslings were randomly assigned to two treatment groups, with 100 birds in each group. As previously reported (Li H et al., 2024), the goslings in the experimental group were subcutaneously injected with 0.2 mL of chorioallantois membrane homogenates containing 2 × 10^−6^ TCID_50_ of the GoAstV strain (GoAstV group), while those in the control group were injected with an equal volume of a saline solution (Control group). All goslings were monitored daily for the occurrence of clinical signs, growth, and mortality.

At 6 days post-inoculation (dpi), six goslings (*n* = 6) were randomly selected from each treatment group. Blood was collected from the wing vein, and the goslings were then slaughtered, weighed, and their liver tissues were collected. The blood samples were immediately transferred to 2 mL Eppendorf tubes and then centrifuged at 2,500 rpm at 4°C for 15 min to separate the serum. The liver tissues were obtained from the same region of each bird. A small square of the liver tissue was fixed in 4% paraformaldehyde for histopathological analysis, while another cube was collected and immediately snap-frozen in liquid nitrogen. All frozen serum and tissue samples were stored at −80°C until further analysis.

### 2.2 Viral loads detection

To detect the copy number of GoAstV, viral RNA (n = 6) was extracted from the liver tissues using the TRIzol reagent (Life Technologies, USA) and normalized to 1 μg of total RNA per sample. Real-time fluorescence quantitative PCR (qRT-PCR) was performed using AceQ qPCR Probe Master Mix (Q112-02, Nanjing, China) and the TaqMan one-step RT-PCR method established in our laboratory (Li et al., 2023), based on the Roche LightCycler 96 real-time PCR system (Roche Diagnostics, Shanghai, China).

### 2.3 Morphological analysis

#### 2.3.1 Hematoxylin–eosin (HE) staining

The histopathological changes in the gosling livers (*n* = 6) were examined using the classical HE staining method. Briefly, the liver tissues fixed in 4% paraformaldehyde were embedded in paraffin blocks and cut into 7–8 μm slices. Afterward, these slices were dewaxed and stained with hematoxylin and eosin at Pinuofei Biological Technology Co., Ltd. (Wuhan, China). Finally, the sections were examined and scanned under a light microscope (Pannoramic MIDI, Hungary) and analyzed using the CaseViewer image viewing software (version 2.5.0).

#### 2.3.2 Oil red O staining

Oil Red O staining was conducted to evaluate lipid deposition in the gosling hepatocytes. In brief, the frozen liver blocks (*n* = 6) were embedded in optimal cutting temperature (OCT) compound and sectioned into 7–8 μm slices. Then, the slices were washed with 1 × PBS and stained with an Oil Red O working solution at Pinuofei Biological Technology Co., Ltd. (Wuhan, China). The stained liver slices were examined and scanned under a light microscope (Pannoramic MIDI, Hungary), and the intracellular lipid content of the gosling livers was further analyzed using Image J (version 1.8.0).

### 2.4 Hepatic and serum parameters measurement

The liver weight was obtained immediately after the goslings were slaughtered, and the liver index was calculated using the following formula: Liver index = [Liver weight (g)/Body weight (g)] × 100. The biochemical parameters, including hepatic alanine transaminase (ALT) and aspartate transaminase (AST), as well as serum superoxide dismutase (SOD), triglycerides (TG), total cholesterol (TC), low-density lipoprotein cholesterol (LDL-C), and high-density lipoprotein cholesterol (HDL-C), were evaluated (*n* = 6) using an automatic analyzer (Hitachi7060, Hitachi, Tokyo, Japan) according to the manufacturer’s protocols.

### 2.5 RNA sequencing analysis and signal pathway validation

RNA sequencing (RNA-seq) and bioinformatics analysis were performed by Gene Denovo Biotechnology Co., Ltd. (Guangzhou, China). Briefly, total RNA was extracted from the liver tissues (*n* = 4) using the TRIzol reagent (Life Technologies, USA). Agarose gel electrophoresis and a NanoDrop microspectrophotometer (Thermo Fisher, Germany) were used to assess the integrity and purity of the total RNA. Subsequently, mRNAs were separated from the total RNA, fragmented, and reverse-transcribed into cDNA. After PCR library enrichment, the sequencing was performed using the Illumina sequencing platform. Quality control and screening of raw data were performed using fastp (version 0.18.0), and data comparison was performed with Bowtie2 (version 2.2.8). Then, the sequences were aligned to the *Anser cygnoides* reference genome (NCBI_GCF_000971095.1) using HISAT (version 2.2.4). Principal component analysis (PCA) analysis, differentially expressed genes (DEGs) screening, Gene Ontology (GO), and Kyoto Encyclopedia of Genes and Genomes (KEGG) enrichment were carried out using StringTie (version 1.3.1), RSEM (version 1.3.3), DESeq2 (version 1.20.0), and R project packages, respectively.

Peroxisome proliferator-activated receptor *α*/*γ* (PPARα/γ) and six DEGs from the lipid metabolism-related pathways were selected to validate the accuracy of the RNA-seq data through qRT-PCR. Briefly, total RNA was extracted from the frozen gosling liver tissues (*n* = 6) using the TRIzol reagent (15,596,026, Invitrogen, CA, USA) and reverse-transcribed into cDNA on an A200 Gradient Thermal Cycler (LongGene, Hangzhou, China) using the PrimeScript® 1st Strand cDNA Synthesis Kit (D6110A, Takara, Dalian, China). Then, the diluted cDNA was used as a template to perform qRT-PCR on the Mx3000P system (Mx3000P, Stratagene, USA) using the TB Green® Premix Ex Taq™ II (TaKaRa, Dalian, China) reaction system, according to the manufacturer’s instructions. Glyceraldehyde-3-phosphate dehydrogenase (GAPDH) was chosen as the internal control, and all the primer sequences are shown in Supplementary Table S1.

### 2.6 Statistical analysis

Statistical comparisons among the groups were performed using the independent samples *t*-test and one-way ANOVA with SPSS 25.0 for Windows. The 2^−ΔΔCt^ method was used to analyze the real-time PCR data. All data were presented in the format of mean ± SEM. Differences with a *p*-value of <0.05 were considered statistically significant.

## 3 Results

### 3.1 Phenotypic changes in the gosling liver after the GoAstV infection

**Figure 1:**
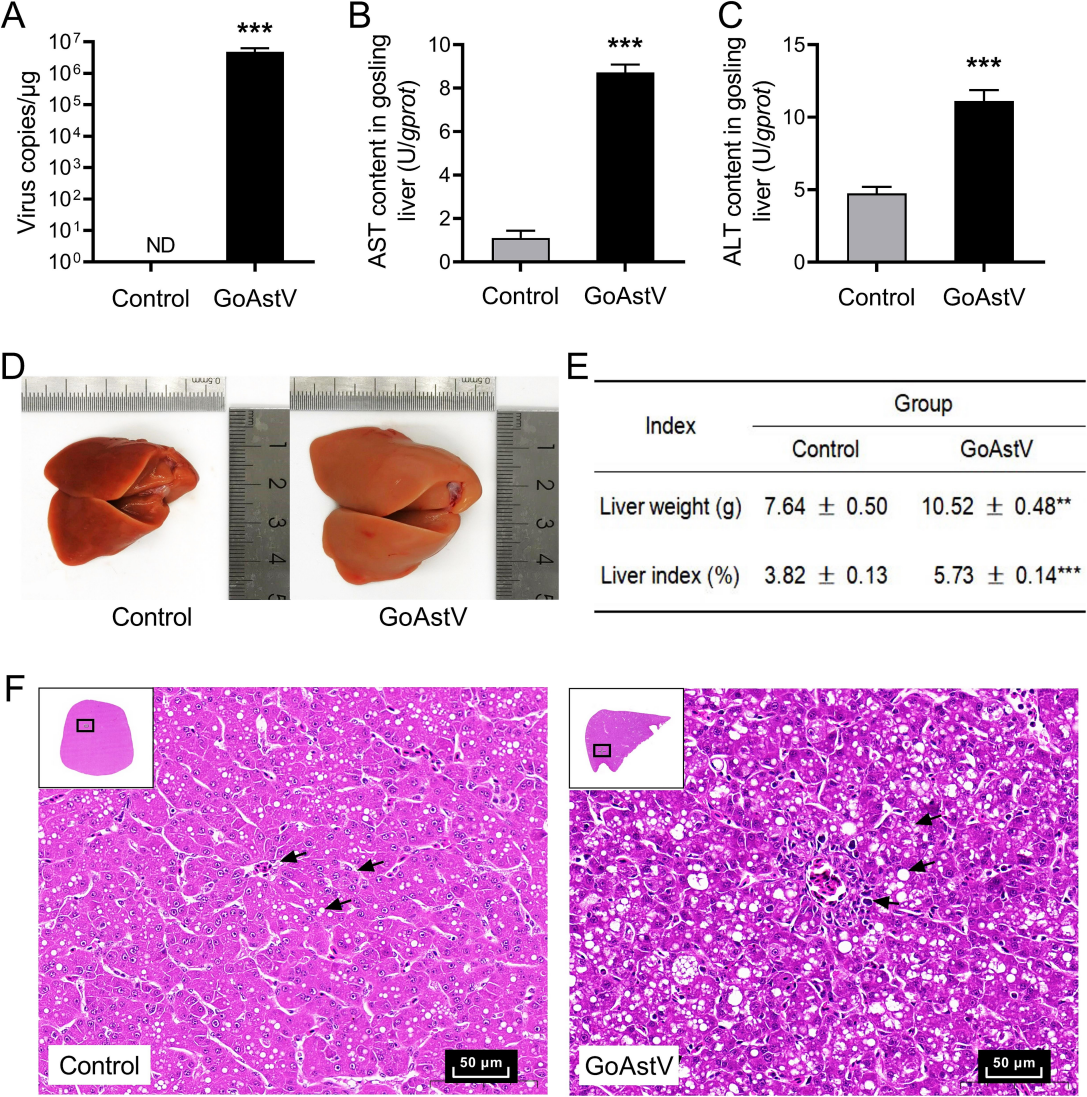
Phenotypic changes in the gosling livers after the GoAstV infection. **(A)** The copy number of GoAstV in the gosling livers. The birds were randomly assigned to two experimental groups, and samples were collected at 6 days post-infection (6 dpi); **(B,C)** The AST and ALT concentrations in the gosling livers; **(D,E)** The alterations in the liver weight and liver index of the gosling livers, along with the representative image (400×); **(F)** Hematoxylin–eosin (H&E) staining of the liver tissues from both experimental groups. The data were expressed as mean ± SEM (*n* = 6). Control group: The birds were subcutaneously injected with 0.2 mL of a saline solution. GoAstV group: The goslings were subcutaneously injected with 0.2 mL of chorioallantois membrane homogenates containing 2 × 10^−6^ TCID_50_ of the GoAstV strain. **p < 0.01 and *** *p* < 0.001, compared to the Control group.

As shown in Figure 1, the GoAstV infection significantly impaired the liver function in the goslings. At the organ level, the viral copies, liver volume, liver weight, and liver index were significantly (*p* < 0.01) increased in the GoAstV group at 6 dpi compared to the Control group (Figures 1A,D,E). At the cellular and molecular levels, swelling degeneration and inflammatory cell infiltration were widely observed in the liver tissues. The arrangement of the hepatic cords was disordered, and the hepatic sinuses were narrowed or completely disappeared following the virus exposure (Figure 1F). At the same time, ALT and AST, two key indicators of liver function impairment, were both significantly (*p* < 0.001) increased in the gosling livers after the GoAstV infection (Figures 1B,C).

### 3.2 Hepatic inflammatory, oxidative, and apoptotic reactions after the GoAstV infection

**Figure 2:**
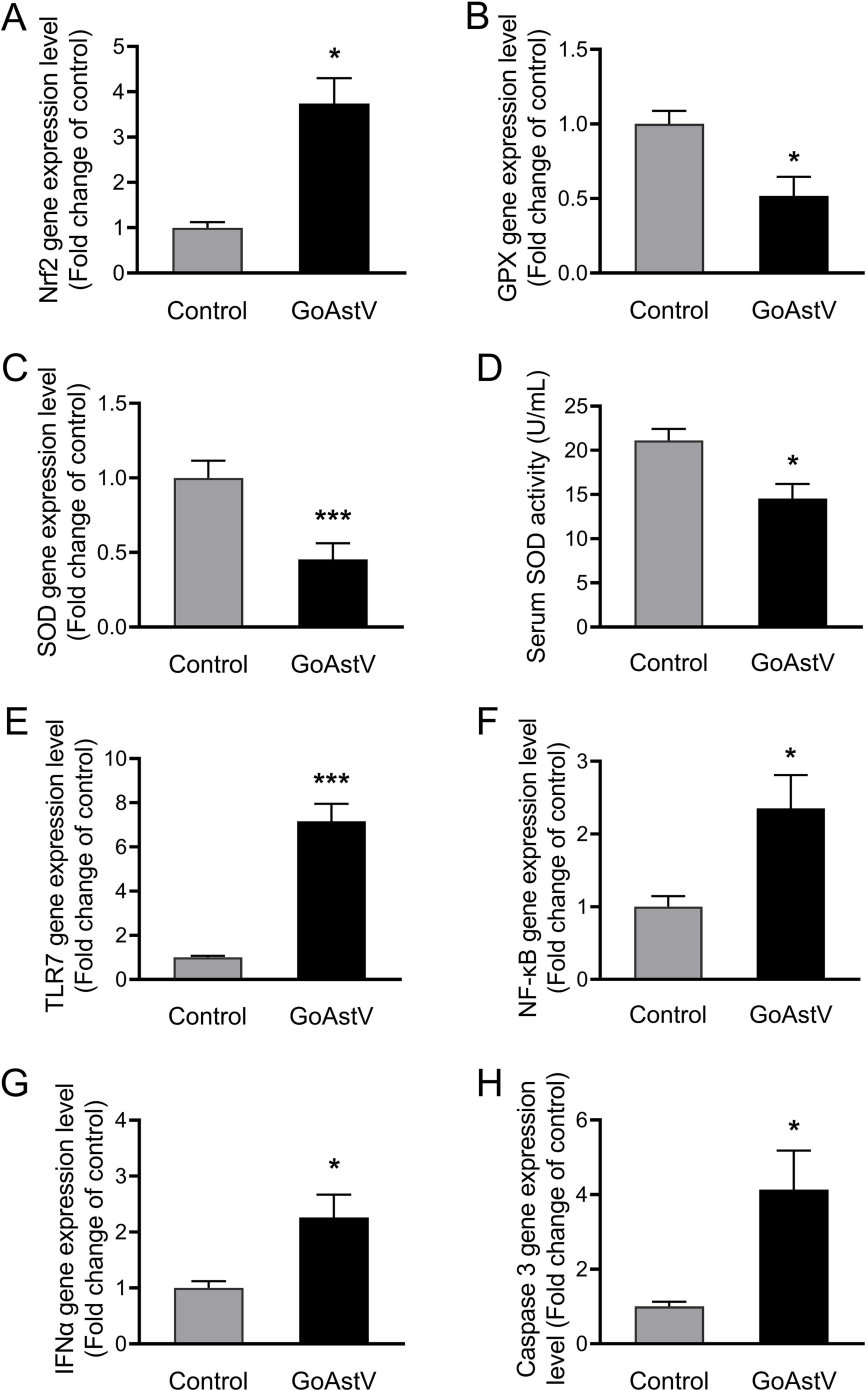
Hepatic inflammatory, oxidative, and apoptotic responses after GoAstV infection. **(A–D)** The expression levels of the cellular oxidation-related genes **(A–C)** and the enzymatic activity of the serum SOD in the goslings **(D)**; **(E–H)** The expression levels of the inflammation- **(E–G)** and cell apoptosis **(H)**-related genes in the gosling livers. The data were expressed as mean ± SEM (*n* = 6). Control: The birds were subcutaneously injected with 0.2 mL of a saline solution. GoAstV: The goslings were subcutaneously injected with 0.2 mL of chorioallantois membrane homogenates containing 2 × 10^−6^ TCID_50_ of the GoAstV strain. *p < 0.05 and *** *p* < 0.001, compared to the Control group.

In addition to the histological changes mentioned above, inflammatory, oxidative, and apoptotic responses were also observed in the hepatocytes after the GoAstV infection. As shown in Figure 2, despite a significant (*p* < 0.05) elevation in *Nrf2* (Figure 2A), the expression levels of the antioxidant genes *GPX* and *SOD,* as well as the enzymatic activity of the serum SOD (*p* < 0.05), were significantly decreased (*p* < 0.05) after the GoAstV injection (Figures 2B–D). On the contrary, the inflammation-related genes, including *TLR7*, *NF-κB*, and *INFα,* as well as the key regulatory gene *Caspase 3* involved in cell apoptosis, were markedly (*p* < 0.05) up-regulated following the GoAstV infection (Figures 2E–H).

### 3.3 Transcription profile and differentially expressed genes annotation

**Figure 3:**
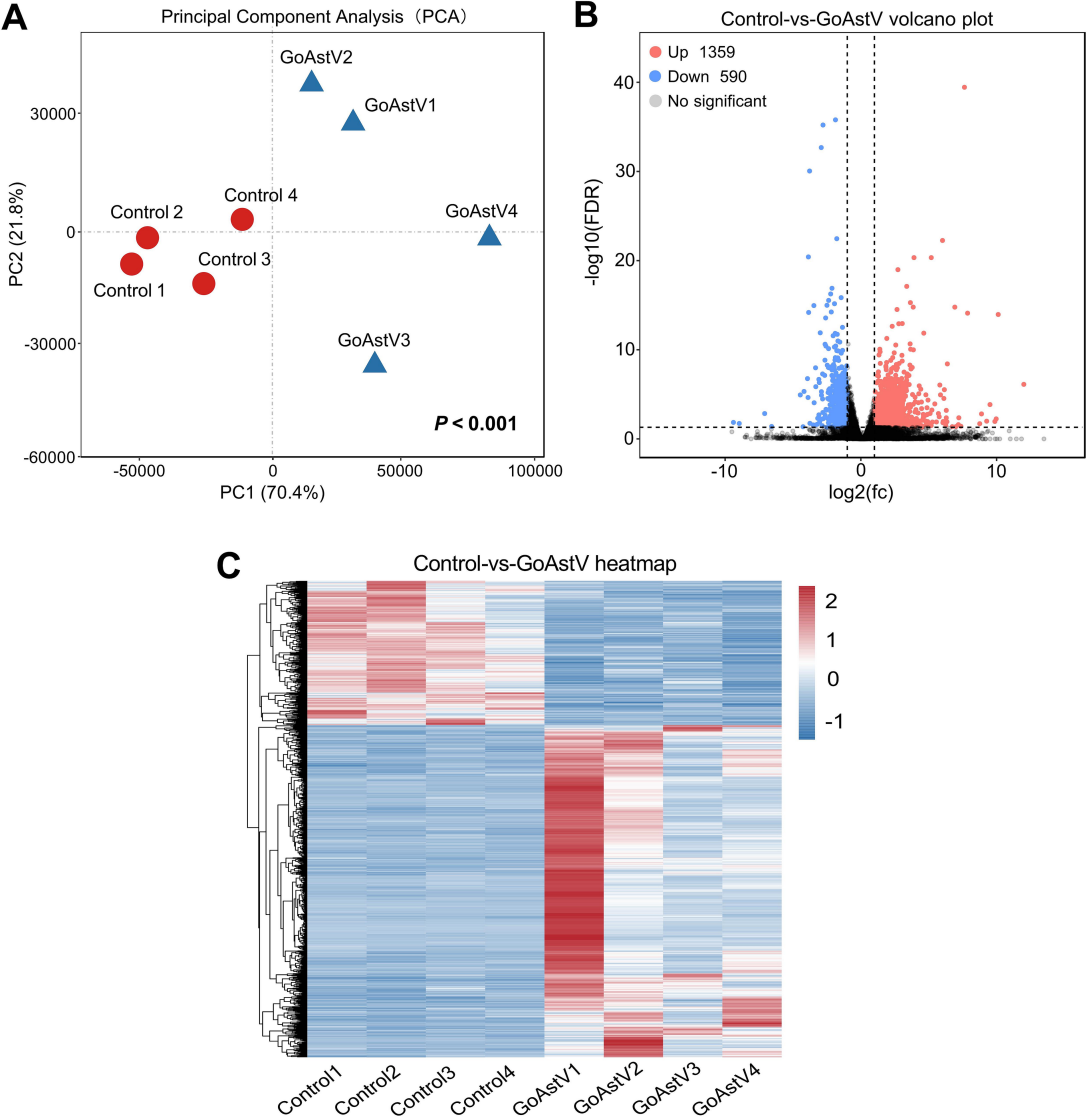
Transcription profile and annotation of the differentially expressed genes in the gosling livers post-GoAstV infection. **(A)** The PCA score plots showing the variance in the genes between the Control and GoAstV groups (*n* = 4); **(B,C)** Representative images of the volcano plot **(B)** and heatmap **(C)** of the DEGs identified from the two experimental groups (*n* = 4). Control group: The birds were subcutaneously injected with 0.2 mL of a saline solution. GoAstV group: The goslings were subcutaneously injected with 0.2 mL of chorioallantois membrane homogenates containing 2 × 10^−6^ TCID_50_ of the GoAstV strain.

Hepatic transcriptome analysis was performed to further explore the potential pathways and target the genes involved in the GoAstV-induced dysfunction of the gosling livers. Using the Illumina HiSeq 6,000 platform, an average of 49,578,084 and 51,148,508 qualified clean reads were obtained in the Control and GoAstV groups, respectively, with an average mapping rate of approximately 83.7% and Q30 ≥ 92.7%, as shown in detail in Supplementary Table S2. The principal component analysis (PCA) plot revealed that although variations existed in the GoAstV group, a clear separation of the samples was found between the two experimental groups, indicating dramatic changes in the gene expression caused by the virus infection (Figures 3A,C). In addition, using DESeq2 (v1.34.0) analysis, a total of 1,949 differentially expressed genes (DEGs) were identified, of which 1,359 DEGs were up-regulated and 590 DEGs were down-regulated compared to the Control group (Figures 3B,C).

### 3.4 GO and KEGG enrichment analysis of the DEGs

**Figure 4:**
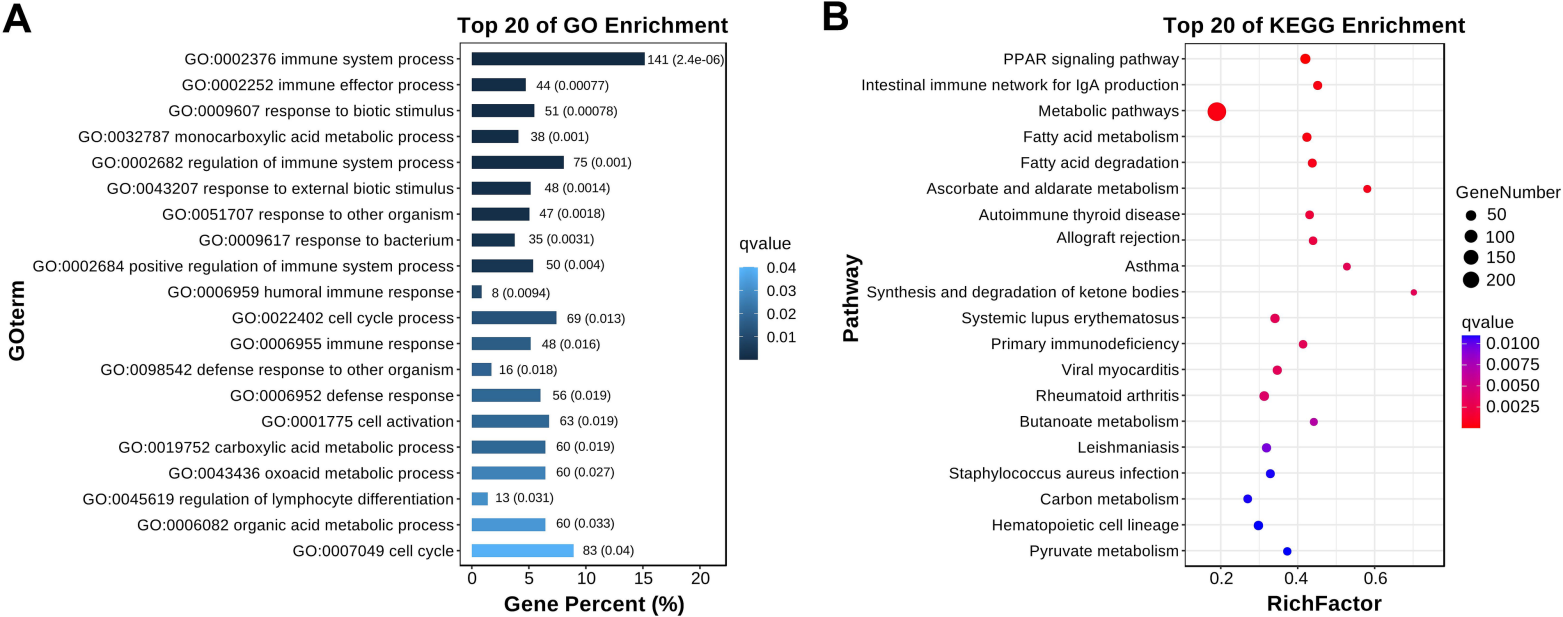
Representative graphs of the GO and KEGG enrichment analyses of the DEGs. Differential metabolites and their set enrichment analysis. **(A)** The top 20 GO terms identified by the GO analysis. The numbers behind each rectangle indicate the count and significance level of the genes involved in these GO terms; **(B)** The top 20 pathways identified by the KEGG enrichment analysis. The abscissa and the size of the bubbles represent the importance and gene number of the pathways in the topology analysis, respectively, and the ordinate and bubble color (from red to blue) denote the significance level of each pathway in the enrichment analysis.

The DEGs were subsequently classified through GO and KEGG pathway enrichment analyses. The results showed that, in the top 20 GO terms, the DEGs were mainly concentrated in organic immune defense functions, such as the immune system process, immune effector process, response to a biotic stimulus, regulation of the immune system process, and material metabolic processes, such as the monocarboxylic acid metabolic process, carboxylic acid metabolic process, and organic acid metabolic process (Figure 4A).

Similar outcomes were found in the KEGG enrichment analysis. As shown in Figure 4B, in the top 20 pathways, the majority of the DEGs were enriched in pathways closely associated with organic immune and metabolic regulation, such as the intestinal immune network for IgA production, autoimmune thyroid disease, primary immunodeficiency, PPAR signaling pathway, metabolic pathway, fatty acid metabolism and degradation, and ascorbate and aldarate metabolism (Figure 4B). The detailed information of the genes involved in each KEGG pathway is listed in Supplementary Table S3.

### 3.5 Alterations in the lipid metabolism after the GoAstV infection

**Figure 5:**
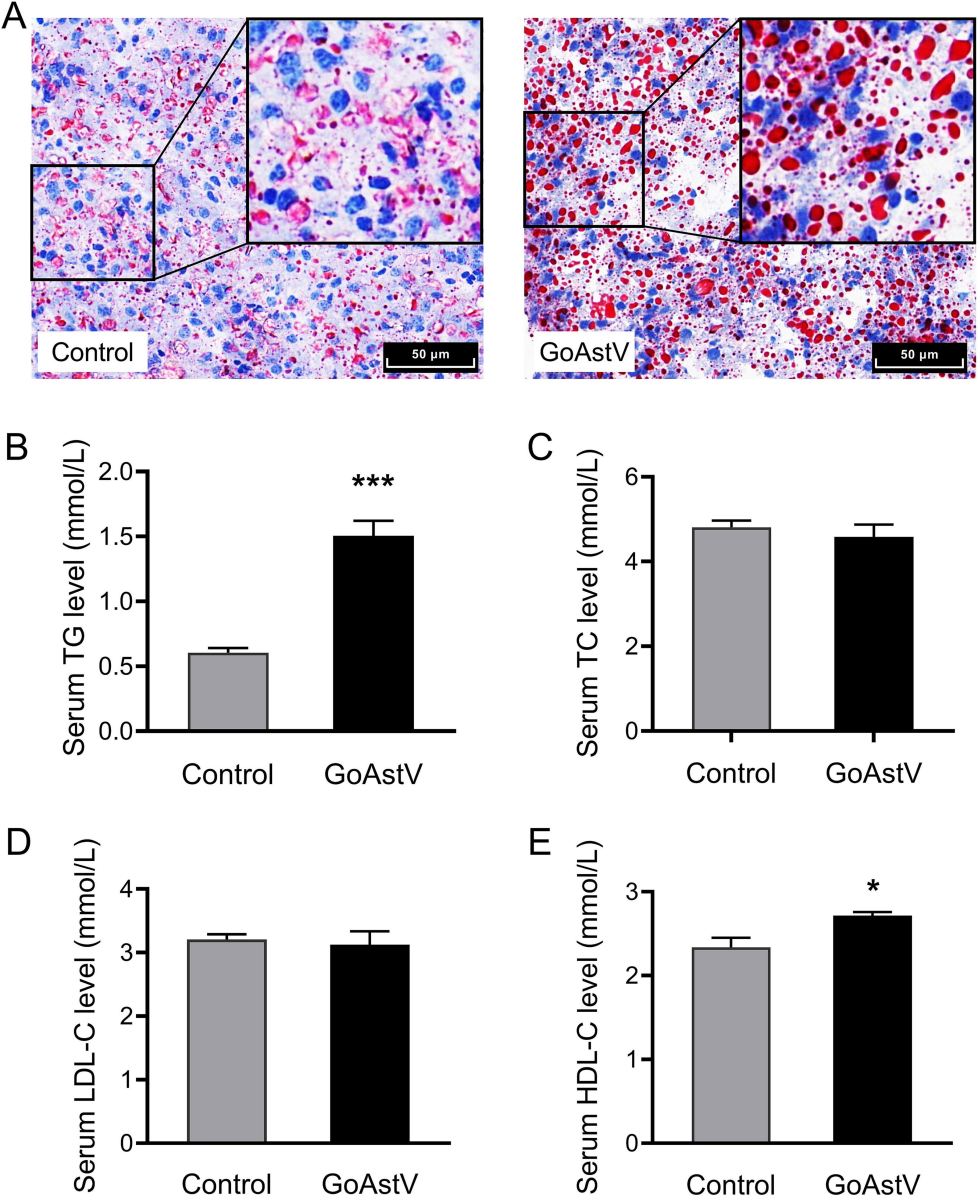
Alterations in the hepatic lipid metabolism in the gosling exposed to GoAstV. **(A)** Representative images of lipid deposition in the gosling hepatocytes by Oil red O staining (400×); **(B–E)** The comparison of the serum concentrations of TG **(B)**, TC **(C)**, LDL-C **(D)**, and HDL-C **(E)** between the two experimental groups. The data were expressed as mean ± SEM (*n* = 6). Control group: The birds were subcutaneously injected with 0.2 mL of a saline solution. GoAstV group: The goslings were subcutaneously injected with 0.2 mL of chorioallantois membrane homogenates containing 2 × 10^−6^ TCID_50_ of the GoAstV strain. *p < 0.05 and ***p < 0.001, compared to the Control group.

As indicated by the KEGG analysis, the most significant DEGs were mainly concentrated in the pathways related to the organic lipid metabolism. Therefore, the lipid metabolic changes in the gosling liver and blood were further examined. The Oil red O staining showed that, compared to the Control group, the number of the stained fat particles increased significantly (*p* < 0.05) in the GoAstV-infected gosling hepatocytes (Figure 5A). In addition, increased fat deposition in the liver of the birds after the virus exposure was confirmed by the significantly (*p* < 0.05) elevated serum free TG and HDL-C concentrations, as shown in Figures 5B–E.

### 3.6 Validation of the gene expression in the lipid metabolism-related pathways

**Figure 6:**
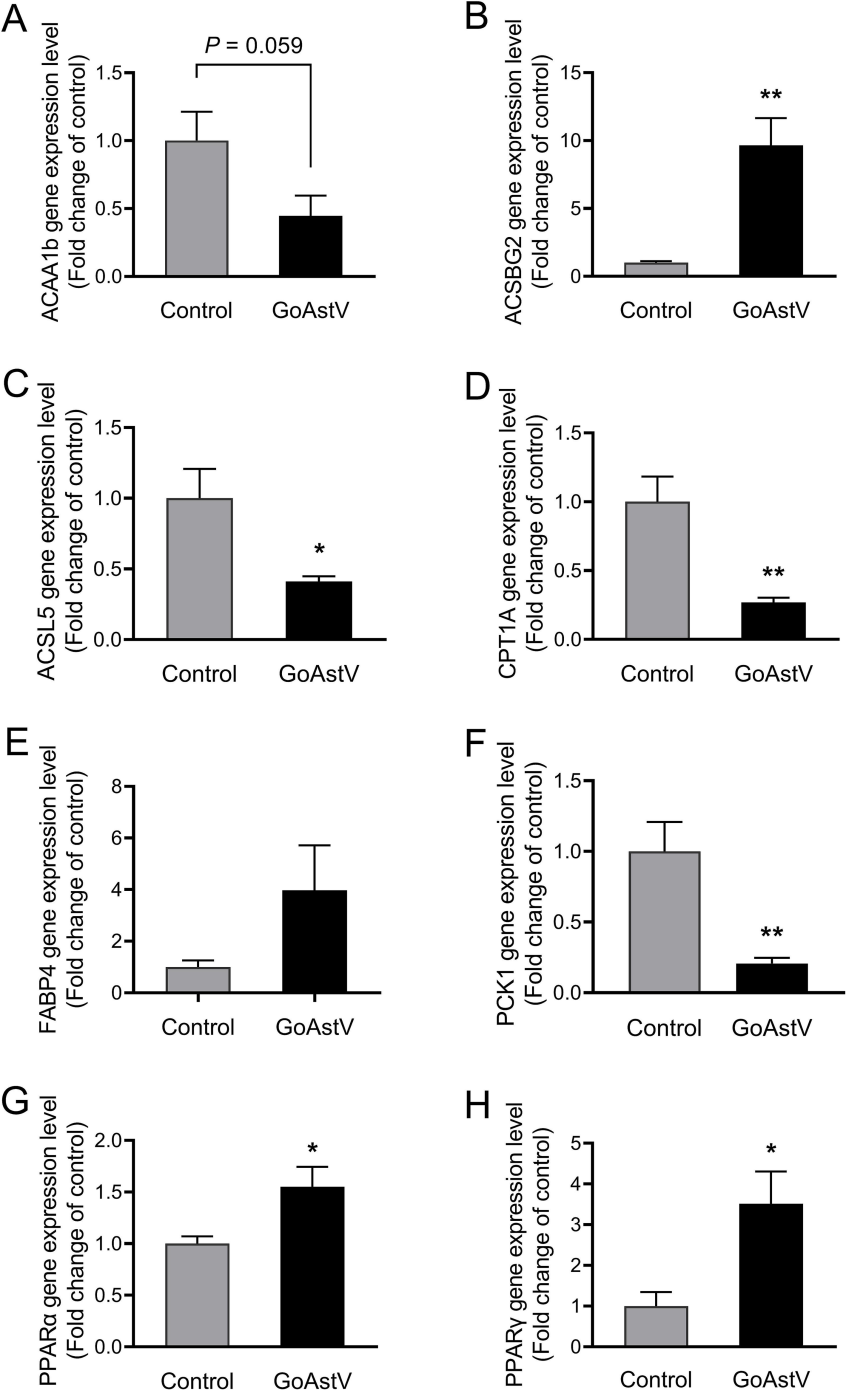
Validation of the gene expression in the lipid metabolism-related pathways. To further verify the accuracy of the RNA sequencing results and explore the underlying mechanism contributing to the altered lipid metabolism in the gosling livers, the expression levels of PPARα, PPARγ, and the six genes randomly selected from the lipid metabolism-related KEGG pathways were assessed using RT-PCR, as shown in **(A–H)**. Control group: The birds were subcutaneously injected with 0.2 mL of a saline solution. GoAstV group: The goslings were subcutaneously injected with 0.2 mL of chorioallantois membrane homogenates containing 2 × 10^−6^ TCID_50_ of the GoAstV strain. *p < 0.05 and ** *p* < 0.01, compared to the Control group.

The expression levels of PPARα and PPARγ, as well as the six genes randomly selected from the lipid metabolism-related pathways, were tested to verify the accuracy of the RNA sequencing results and disclose the underlying mechanism contributing to the altered lipid metabolism in the gosling livers. As shown in Figure 6, except for *ACAA1b* and *FABP4*, the expression patterns of *ACSBG2*, *PPARα*, *PPARγ, ACSL5*, *CPT1A*, and *PCK1* were fully consistent with those from the RNA-seq data. Specifically, the first three genes were markedly (*p* < 0.05) up-regulated, while the last three were down-regulated in the GoAstV group compared to the Control group.

## 4 Discussion

Goose astrovirus (GoAstV) is a newly discovered RNA virus that causes organic dysfunction and urate deposition in the joints and viscera of goslings under 3 weeks of age (An et al., 2020; Zhu and Sun, 2022). This virus not only has a pathogenic rate as high as 80% and a fatality rate of 50% in goslings (Wei et al., 2020b; Yang et al., 2018) but also, due to genetic variations generated during RNA replication, has the potential to cross the species barrier (Holland et al., 1982; Wei et al., 2020a), thereby causing significant economic losses to the poultry-breeding industry in China.

Studies have shown that GoAstV leads to organic lesions and dysfunctions mainly by triggering cellular inflammatory responses, oxidative stress, cell apoptosis, and host immunosuppression (Ding et al., 2021; Jin et al., 2018; Yin et al., 2021). Especially in the liver, which is not only the most important organ for material and energy metabolism but also the primary site of uric acid (UA) production in poultry (Dalbeth et al., 2016; Maiuolo et al., 2016). For example, Yin et al. (2021) reported that inoculating 1-day-old goslings with an SDPY astrovirus strain resulted in the majority of the goslings dying between 6 and 8 dpi. At the same time, obvious lesions and urate precipitation, as well as marked increases in serum ALT, AST, UA, and urea nitrogen (UN) levels, were found in the gosling livers. However, the functional alterations and their regulatory mechanisms in the gosling liver in response to GoAstV infection remain unclear. Therefore, in this study, morphological and RT-PCR techniques were first used to detect changes in the hepatic phenotype after the GoAstV infection. We found that, in accordance with previous research (Ding et al., 2021; Li Y et al., 2024; Ma et al., 2021; Xu et al., 2023), the structure and function of the gosling livers were visibly altered by the virus infection, as revealed by the histological alterations, such as the liver index, hepatocellular morphology, hepatic cords and sinuses, abnormal expression patterns of cellular antioxidant, inflammation, and apoptosis-related genes. However, in contrast to previous reports, we did not observe apparent urate crystallization or deposition in the gosling liver tissues. This may be due to differences in the virus strain, dosage, or infection duration used in this study (An et al., 2020; Ma et al., 2021).

RNA-seq analysis of the liver tissues was performed to further investigate how GoAstV induced damage to the gosling livers. A total of 1,949 differentially expressed genes (DEGs) were identified between the two experimental groups. The GO and KEGG analysis showed that the majority of these DEGs were enriched in GO terms related to immune defense and substance metabolism, as well as their corresponding regulatory pathways, such as the PPAR signaling pathway, intestinal immune network for IgA production, and fatty acid metabolism and degradation. This suggested that the GoAstV-induced changes in the gosling liver function were mainly concentrated on lipid metabolism. This finding was further confirmed by the Oil red O staining and blood biochemical analysis, which showed significant increases in the lipid droplet deposition and the serum concentrations of TG and HDL-C in the GoAstV-infected hepatocytes, compared to the Control group. Similarly, the expression of eight genes selected from the lipid metabolism-related pathways, such as *ACSBG2,* which is involved in lipogenesis, and *ACSL5*, *CPT1A*, and *PCK1,* which are correlated with fatty acid degradation, further demonstrated the potential contributions of these pathways to the lipid metabolism regulation in the gosling livers. However, surprisingly, we also found that the genes PPARα and PPARγ, involved in fatty acid oxidation and utilization and fatty acid transportation, respectively (Nguyen et al., 2008), were both significantly up-regulated in our study. Based on previous research, we suspect that two factors may explain this finding: on the one hand, in addition to lipid metabolism regulation, PPARα and PPARγ play crucial roles in various biological processes, such as organic growth and development, cell fate determination, and inflammatory responses (Berger and Moller, 2002; Feige et al., 2006). Therefore, the elevation of these two genes might have resulted from the GoAstV-induced inflammatory reactions we mentioned before. On the other hand, excessive lipid accumulation in gosling liver tissues triggers a negative feedback response in the animal. Therefore, this might have compensatively caused the up-regulation of PPARα and PPARγ (Ru and Guo, 2017).

**Figure 7:**
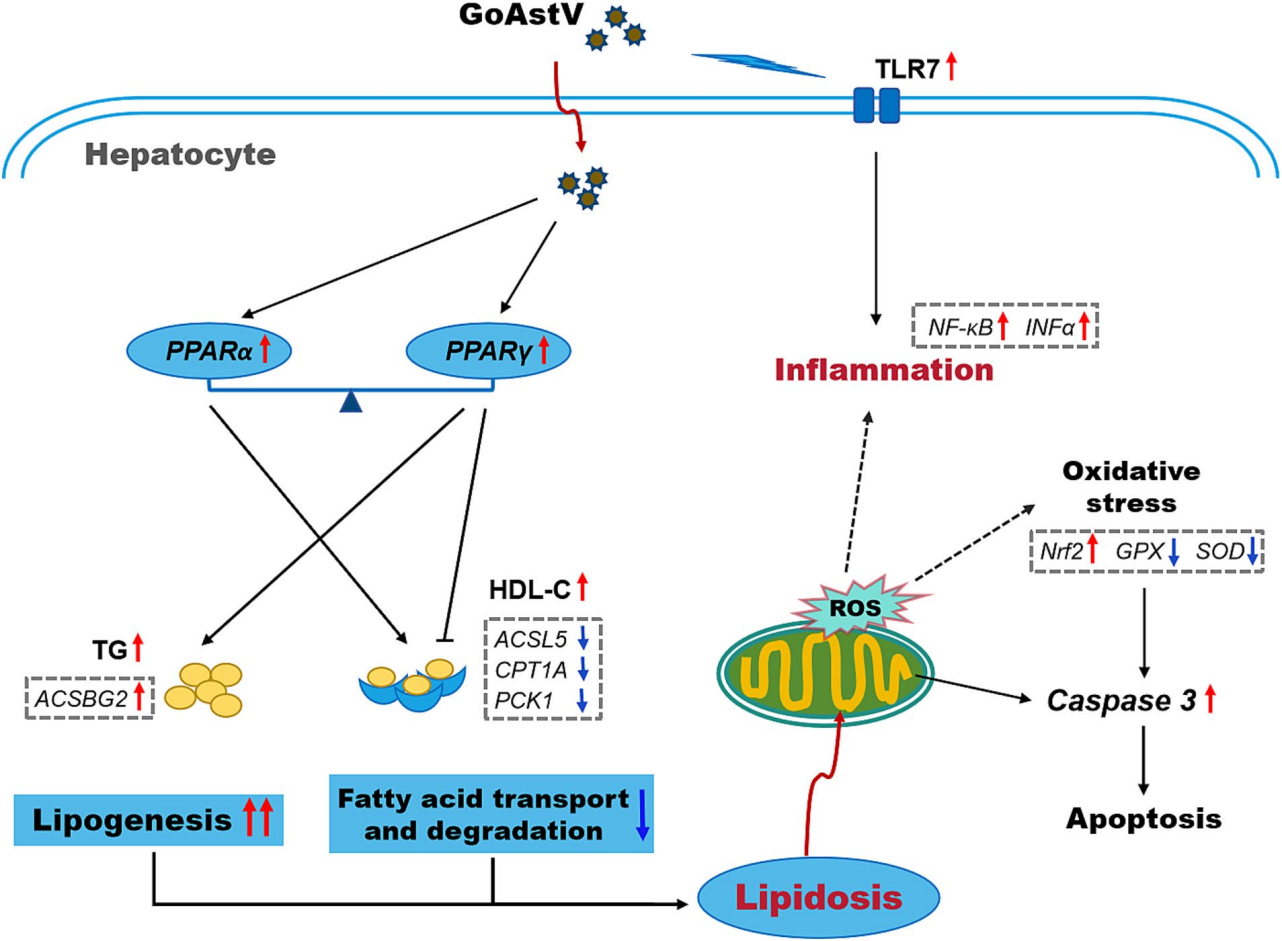
A simplified schematic of the potential mechanisms underlying the GoAstV infection-induced hepatic inflammation and lipid deposition in the goslings.

In conclusion, by combining morphological, biochemical, and RNA-seq analyses, the present study demonstrated that GoAstV infection not only causes significant impairments in the structural and immune functions of gosling livers but also induces excessive lipid deposition in hepatocytes by interfering with gene transcription in lipid metabolism-related signaling pathways (Figure 7). These findings, to a certain extent, expand our understanding of the negative effects of GoAstV on goslings and provide new insights and experimental foundations for addressing GoAstV-induced liver dysfunction in the poultry industry.

## Data Availability

The original contributions presented in the study are publicly available. This data can be found at https://www.ncbi.nlm.nih.gov/sra/PRJNA1219335 with the BioProject accession number: PRJNA1219335.
