# Supplementary material for: Effects and potential pathways of goose astrovirus infection on gosling hepatic lipid metabolism
Source: Front Microbiol. 2025 Feb 25;16:1531373. doi: 10.3389/fmicb.2025.1531373 (PMC11893818; doi:10.3389/fmicb.2025.1531373)
Supplement: Supplementary file 1 [file Table_1.docx]

Table S1 Primer sequences for qRT-PCR

| Name | Sequences (5’→3’) | GenBank No. |
| --- | --- | --- |
| TLR7 | F：CCGACCTTCAGTTAATAGGGA  R：CCTGACAAGTTGAGGCAT | XM_013191543.2 |
| IFNα | F：CAGCACCACATCCACCAC  R：TACTTGTTGATGCCGAGGT | XM_048048544.1 |
| NF-κB | F：GGATGAGGTCTACTTGCTGTG  R：CGTTCTCATCATCCTCGTAGAAG | XM_048069036.1 |
| Caspase 3 | F：CTGGTATTGAGGCAGACAGTGG  R：CAGCACCCTACACAGAGACTGAA | XM_048078360.1 |
| Nrf2 | F：CGCCTTGAAGCTCATCTCAC  R：TTCTTGCCTCTCCTGCGTAT | XM_013171581.2 |
| GPX | F：GCAAGGGGTACAAGCCCAACT  R：GATGATGTACTGCGGGTTGGTC | XM_013201826.2 |
| SOD | F：AAATGGGTGTACCAGCGCAG  R：TCTTCTATTTCTACTTCTGCCACTCC | XM_013192917.2 |
| ACAA1b | F：AAGCCAGGCTGTGTACTGTG  R：CCATGCCAGTTCCAATGCAC | XM_013182449.2 |
| ACSBG2 | F：TACCTCCTCCCTCCAGTGTG  R：GCTTTATCCACTGGCCACCT | XM_048054083.1 |
| ACSL5 | F：TTCCTCCCGCTGACTTGAAC  R：TCCAGAAGCATGCAGTCCTC | XM_013174554.2 |
| CPT1A | F：GCATTGACCGCCATCTGTTC  R：GCCAGCATCTCAGGGTTCTT | XM_048058716.1 |
| FABP4 | F：ATGAAAGAGCTGGGTGTGGG  R：TGTCATCTGCTGTGGTCTCA | XM_013172551.2 |
| PCK1 | F：TTACCCAGGGGGATCTGGAG  R：AGAGCCAACCAGCAGTTCTC | XM_013190722.2 |
| PPARα | F：ATCTATCCCTGGCTTCTCCA  R：AGCATCCCATCCTTGTTCATT | XM_013184640.2 |
| PPARγ | F：CCTCCTTCCCCACCCTATT  R：CTTGTCCCCACACACACGA | XM_013187709.2 |
| GAPDH | F：CTGATGCTCCCATGTTCGTG  R：CCACGATGCCAAAGTTGTCA | XM_013199522.2 |
