# Supplementary material for: Effects and potential pathways of goose astrovirus infection on gosling hepatic lipid metabolism
Source: Front Microbiol. 2025 Feb 25;16:1531373. doi: 10.3389/fmicb.2025.1531373 (PMC11893818; doi:10.3389/fmicb.2025.1531373)
Supplement: Supplementary file 2 [file Table_2.docx]

Table S2 Summary of the RNA sequencing (RNA-seq) data and the reads mapping

| Samples | Raw reads | Clean reads (%) | Mapped rate (%) | Q20 bases rate (%) | Q30 bases rate (%) | GC (%) |
| --- | --- | --- | --- | --- | --- | --- |
| Control 1 | 47325242 | 47057804 (99.43%) | 41134669 (87.64%) | 97.47% | 92.91% | 49.03% |
| Control 2 | 46685346 | 46458712 (99.51%) | 40510117 (87.46%) | 97.45% | 92.79% | 48.97% |
| Control 3 | 48485782 | 48075482 (99.15%) | 40759528 (85.00%) | 97.22% | 92.45% | 50.04% |
| Control 4 | 55815966 | 55423342 (99.30%) | 46828675 (84.83%) | 97.51% | 93.00% | 50.21% |
| GoAstV 1 | 57194160 | 56674376 (99.09%) | 45023304 (80.13%) | 97.11% | 92.25% | 51.32% |
| GoAstV 2 | 49203732 | 48731850 (99.04%) | 39089652 (80.74%) | 97.07% | 92.19% | 51.52% |
| GoAstV 3 | 49269706 | 48881034 (99.21%) | 40790558 (83.83%) | 97.45% | 92.98% | 51.62% |
| GoAstV 4 | 48926436 | 48552854 (99.24%) | 38716633 (80.21%) | 97.40% | 92.75% | 50.83% |
